# Supplementary material for: Endocytosis inhibitors block SARS-CoV-2 pseudoparticle infection of mink lung epithelium
Source: Front Microbiol. 2023 Nov 14;14:1258975. doi: 10.3389/fmicb.2023.1258975 (PMC10682793; doi:10.3389/fmicb.2023.1258975)
Supplement: Supplementary file 1 [file Data_Sheet_1.docx]

Supplementary Material

SARS-CoV-2 Pseudoparticles Enter the Mink Lung Epithelium via Endocytosis

Ann Song, Rattapol Phandthong, Prue Talbot^*^

*** Correspondence:** Prue Talbot: talbot@ucr.edu

# Supplementary Figure


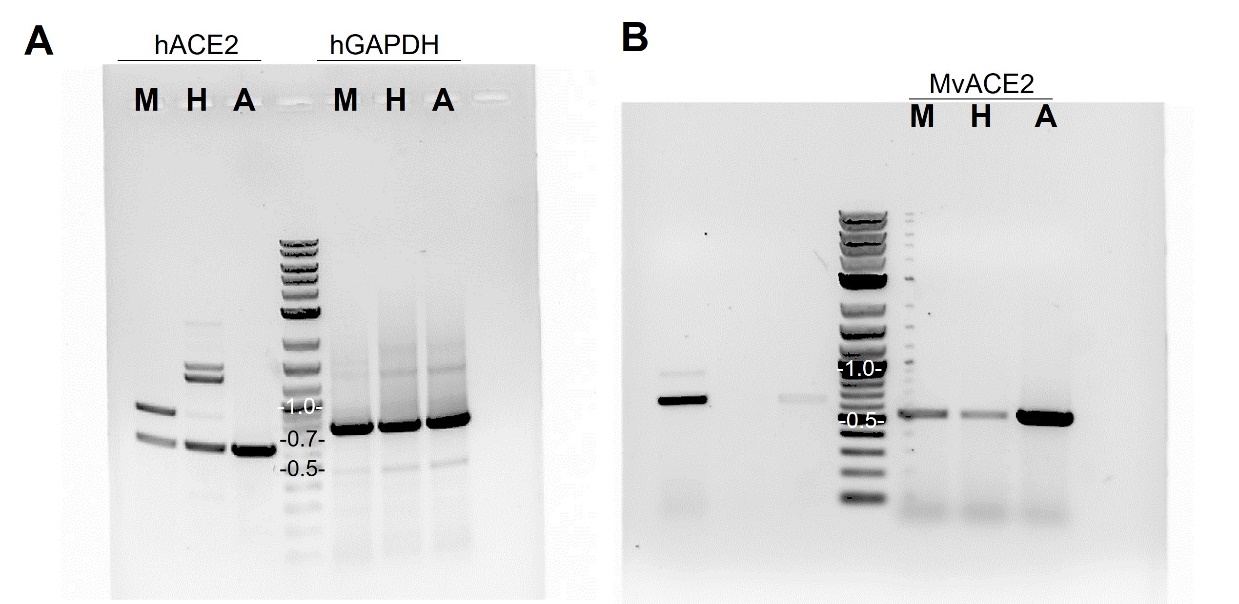


**Supplementary Figure 1.** RT-PCR analysis of ACE2 expression in Mv1Lu cells. A. hACE2 = human ACE2 primers (0.647 kbp); hGAPDH = GAPDH primers (housekeeping control, 0.791 kbp) B. MvACE2 primers = mink ACE2 primers (0.546 kbp). The first three lanes are not part of this experiment. M = RNA extracted from Mv1Lu; H = RNA extracted from HEK 293T; A = RNA from HEK 293T cells stably overexpressing ACE2. Mv1Lu cells expressed the ACE2 transcript using both primer sets.
